# DNA damage-induced S and G2/M cell cycle arrest requires mTORC2-dependent regulation of Chk1

**DOI:** 10.18632/oncotarget.2813

**Published:** 2014-11-15

**Authors:** Jogitha Selvarajah, Androulla Elia, Veronica A. Carroll, Abdeladim Moumen

**Affiliations:** ^1^ Cardiovascular and Cell Sciences Research Institute, St George's University of London, Cranmer Terrace, UK; ^2^ Division of Medical Biotechnology, MAscIR Institution, Rabat, Morocco

**Keywords:** mTORC1, mTORC2, Chk1, DNA damage response, cell cycle arrest, breast cancer

## Abstract

mTOR signalling is commonly dysregulated in cancer. Concordantly, mTOR inhibitors have demonstrated efficacy in a subset of tumors and are in clinical trials as combination therapies. Although mTOR is associated with promoting cell survival after DNA damage, the exact mechanisms are not well understood. Moreover, since mTOR exists as two complexes, mTORC1 and mTORC2, the role of mTORC2 in cancer and in the DNA damage response is less well explored. Here, we report that mTOR protein levels and kinase activity are transiently increased by DNA damage in an ATM and ATR-dependent manner. We show that inactivation of mTOR with siRNA or pharmacological inhibition of mTORC1/2 kinase prevents etoposide-induced S and G2/M cell cycle arrest. Further results show that Chk1, a key regulator of the cell cycle arrest, is important for this since ablation of mTOR prevents DNA damage-induced Chk1 phosphorylation and decreases Chk1 protein production. Furthermore, mTORC2 was essential and mTORC1 dispensable, for this role. Importantly, we show that mTORC1/2 inhibition sensitizes breast cancer cells to chemotherapy. Taken together, these results suggest that breast cancer cells may rely on mTORC2-Chk1 pathway for survival and provide evidence that mTOR kinase inhibitors may overcome resistance to DNA-damage based therapies in breast cancer.

## INTRODUCTION

Mammalian target of rapamycin (mTOR) is a serine-threonine kinase of the phosphoinositide 3-kinase-related kinase (PIKK) family which plays a central role in cell growth and it is commonly dysregulated in cancer [1-6]. Other members of this family include ATM, ATR and DNA-PKcs, which have well established roles in DNA damage response signalling. mTOR is the catalytic component of two functionally distinct complexes, mTORC1 and mTORC2. mTORC1 is composed of mTOR, Raptor, LST8/GβL, PRAS40 and DEPTOR and its activity is stimulated by growth factor signals to regulate protein synthesis through 4E-BP1/2 and the S6 kinases, S6K1 and S6K2 [1, 7]. By contrast, mTORC2, which comprises mTOR, Rictor, LST8/GβL, DEPTOR, SIN1 and PRR5 [1], regulates cytoskeletal organization [8, 9] and has a role in phosphorylation of AGC family members including PKC, Akt and SGK to promote cell survival and cell cycle progression [10-12].

Apart from regulating cell growth signalling, mTOR also responds to numerous cell stresses including nutrient and energy availability, as well as genotoxic stress, in order to promote cell survival [1]. However, how mTOR detects DNA damage and signals this to the DNA repair, cell cycle and cell death machineries is still poorly understood. While there is evidence that DNA damage eventually leads to mTORC1 inhibition through p53-dependent mechanisms [13, 14], there are also an increasing number of reports demonstrating that mTORC1 positively regulates p53, [15-18] and that both mTORC1 and mTORC2 pathways are activated following DNA damage [16, 19-21]. Recently, two groups have identified that mTORC1 regulates the DNA damage response through the upregulation of FANCD2 gene expression, a key protein involved in the repair of DNA double-strand breaks [22, 23].

In this study we investigated how mTOR signals to the cell machinery to promote cell survival following DNA damage. We found that both mTORC1 and mTORC2 activities are transiently increased following DNA damage. Inactivation of mTOR, with siRNA or an mTORC1/2 kinase inhibitor, prevented DNA damage induced S and G2/M cell cycle arrest as well as Chk1 activation, demonstrating a requirement of mTOR for cell survival by establishing efficient cell cycle arrest. Furthermore, we show that ablation of mTORC2 prevents Chk1 activation and augments DNA damage-induced cell death, suggesting that breast cancer cells may rely on the mTORC2-Chk1 pathway for survival.

## RESULTS

### mTOR regulation in response to etoposide-induced DNA damage

**Figure 1:**
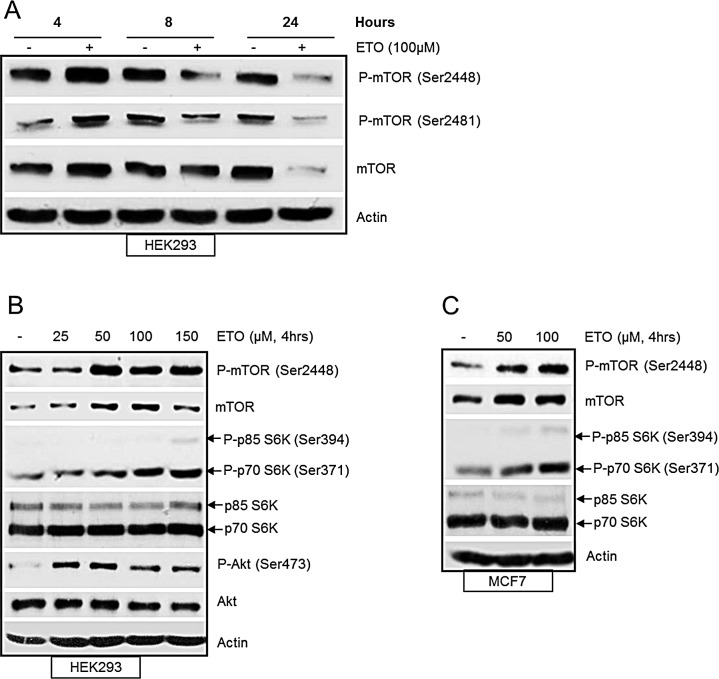
(A) Etoposide-induced DNA damage transiently increases mTOR. HEK293 cells were treated with 100 μM of etoposide for 4, 8 and 24 hrs. Whole-cell lysates were assayed by western blot for mTOR and phosphorylated-mTOR (Ser2448 and Ser2481). Actin was used as loading control. (B) DNA damage-induced increase in mTOR activity is concentration dependent. HEK293 cells were treated with increasing concentrations of etoposide for 4 hrs. Whole-cell lysates were assayed by western blot for mTOR, phosphorylated mTOR (Ser2448), Akt and phosphorylated Akt (Ser473), p70S6K and phosphorylated p70S6K (Ser371). Actin was used as loading control. (C) DNA-damage induced increase in mTOR activity in breast cancer cells. MCF7 cells were treated with 50 and 100 μM etoposide for 4 hrs. Whole-cell lysates were assayed by western blot for mTOR, phosphorylated mTOR (Ser2448), p70S6K and phosphorylated p70S6K (Ser371). Actin was used as loading control.

To examine the regulation of mTOR in response to DNA damage, HEK293 cells were treated with etoposide, a topoisomerase II inhibitor which induces double strand DNA breaks. The level of total mTOR and phosphorylation at Ser2448 and Ser2481 were analysed by western blot at 4, 8 and 24 hrs following etoposide treatment (Figure 1A). We observed a transient increase in total mTOR and phosphorylation at Ser2448 and Ser2481 at 4 hrs after etoposide-induced DNA damage. Total mTOR and phosphorylation levels were eventually decreased at 24 hrs after DNA damage, in agreement with previous reports [13, 14]. We further explored the DNA damage-induced transient increase in mTOR and found a concentration dependent increase in mTOR activity at 4 hrs. mTOR protein level and phosphorylation at Ser2448 were induced by increasing concentrations of etoposide (Figure 1B). In addition, phosphorylation of p70S6K and Akt, downstream targets of mTORC1 and mTORC2 respectively were also increased (Figure 1B). Similar results were observed for MCF7 breast cancer cells with a concentration-dependent increase of phosphorylated mTOR and p70S6K as well as total levels of mTOR (Figure 1C). Collectively these results demonstrate that mTOR protein and its kinase activity are increased in response to early DNA damage.

### mTOR is induced by etoposide in an ATM and ATR-dependent, and p53-independent manner

**Figure 2:**
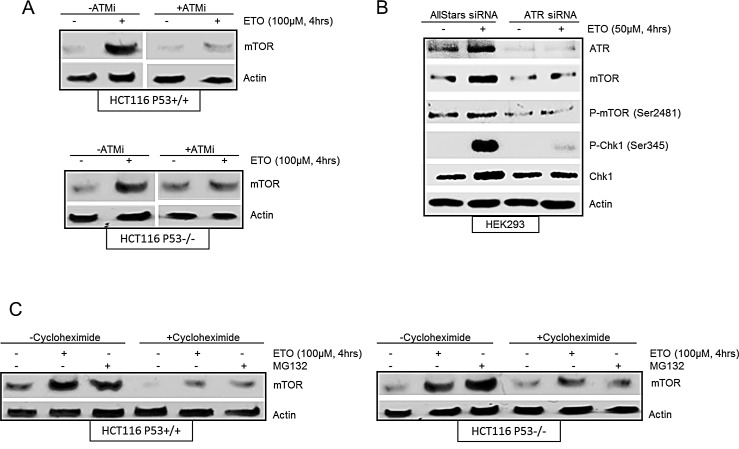
(A) Etoposide induced increase in mTOR is ATM-dependent and p53-independent. HCT116 p53^+/+^ cells and HCT116 p53^−/−^ cells were pre-treated in the absence or presence of 10 μM ATM inhibitor (ATMi) for 1 hr before incubation with 100 μM etoposide for 4 hrs. Whole-cell lysates were assayed by western blot for mTOR. Actin was used as a loading control. (B) Etoposide induced increase in mTOR is ATR-dependent. HEK293 cells were transiently transfected with AllStars siRNA control duplexes or ATR siRNA for 72 hrs. 100 μM of etoposide was added at 4 hrs prior to the end of 72 hrs incubation period. Whole-cell lysates were assayed by western blot for ATR, mTOR and phosphorylated mTOR (Ser2481), Chk1 and phosphorylated Chk1 (Ser345). Actin was used as loading control. (C) mTOR accumulation induced by etoposide is stabilisation. HCT116 p53^+/+^ cells (left panels) and HCT116 p53^−/−^ cells (right panels) were pre-treated in the absence or presence of 10 μM cycloheximide for 1 hr before incubation with either 10 μM of MG-132 or 100 μM of etoposide for a further 4 hrs. Whole-cell lysates were assayed by western blot for mTOR. Actin was used as a loading control.

Previous studies have demonstrated intricate signalling between the mTOR and p53 pathways in response to DNA damage [13, 14]. A number of reports have described p53-mediated down regulation of mTOR signalling [13]. In addition, it has also been shown that increased mTOR activity positively regulates p53 accumulation and function [16, 17, 24, 25]. We were interested in the role of p53 in etoposide-induced increase of mTOR and therefore assessed isogenically matched p53^+/+^ and p53^−/−^ HCT116 cells. Etoposide-induced accumulation of mTOR was observed in both p53^+/+^ and p53^−/−^ cells indicating that mTOR upregulation following early DNA damage was not dependent on p53 (Figure 2A). Next we assessed the role of key regulators of the DNA damage response, ATM and ATR, on etoposide-induced increase in mTOR. An ATM-specific inhibitor was used to establish whether DNA damage-induced transient upregulation of mTOR was dependent on ATM. The transient increase in mTOR following 4 hrs of etoposide treatment was suppressed in the presence of the ATM inhibitor in both p53^+/+^ and p53^−/−^ HCT116 cells (Figure 2A). p53 is a well-studied target of ATM which was monitored by western blot to confirm that the ATM inhibitor was effective (Supplementary Figure 1). These results are consistent with a previous report demonstrating a requirement of ATM for the initial transient increase in protein synthesis induced by DNA damage that was mediated by mTORC1 [26]. In addition, we downregulated ATR using siRNA in HEK293 cells to determine whether etoposide induction of both mTOR protein and phosphorylation at Ser2481 were dependent on ATR (Figure 2B). To ensure that ATR siRNA had sufficiently suppressed ATR activity, phosphorylation of Chk1 (Ser345), a well-known substrate of ATR, was monitored by western blot (Figure 2B). Taken together, our results show that etoposide-induced increase in mTOR is independent of p53, but dependent on ATM and ATR activity.

In order to explore the mechanism of etoposide-induced increase in mTOR protein level, HCT116 p53^+/+^ and p53^−/−^ cells were either treated with cycloheximide, an inhibitor of protein synthesis, or the proteasome inhibitor, MG-132 (Figure 2C). Incubation of cells with cycloheximide alone resulted in inhibition of mTOR protein suggesting a requirement for ongoing protein synthesis to maintain basal mTOR levels. However, the etoposide-mediated increase in mTOR protein accumulation was still observed in both p53^+/+^ and p53^−/−^ HCT116 cells in the presence of cycloheximide, indicating that etoposide-mediated increase in mTOR was unlikely due to increased protein synthesis. We next investigated the effect of MG-132 on the level of mTOR in HCT116 cells. Treatment of cells with MG-132 for 4 hrs led to an accumulation of mTOR protein similar to that observed for etoposide treatment (Figure 2C), either in the absence or presence of cycloheximide, further suggesting that etoposide-mediated upregulation of mTOR was not dependent on protein synthesis, but rather due to stabilization of mTOR.

### mTOR is required for efficient DNA damage-induced cell cycle arrest

**Figure 3:**
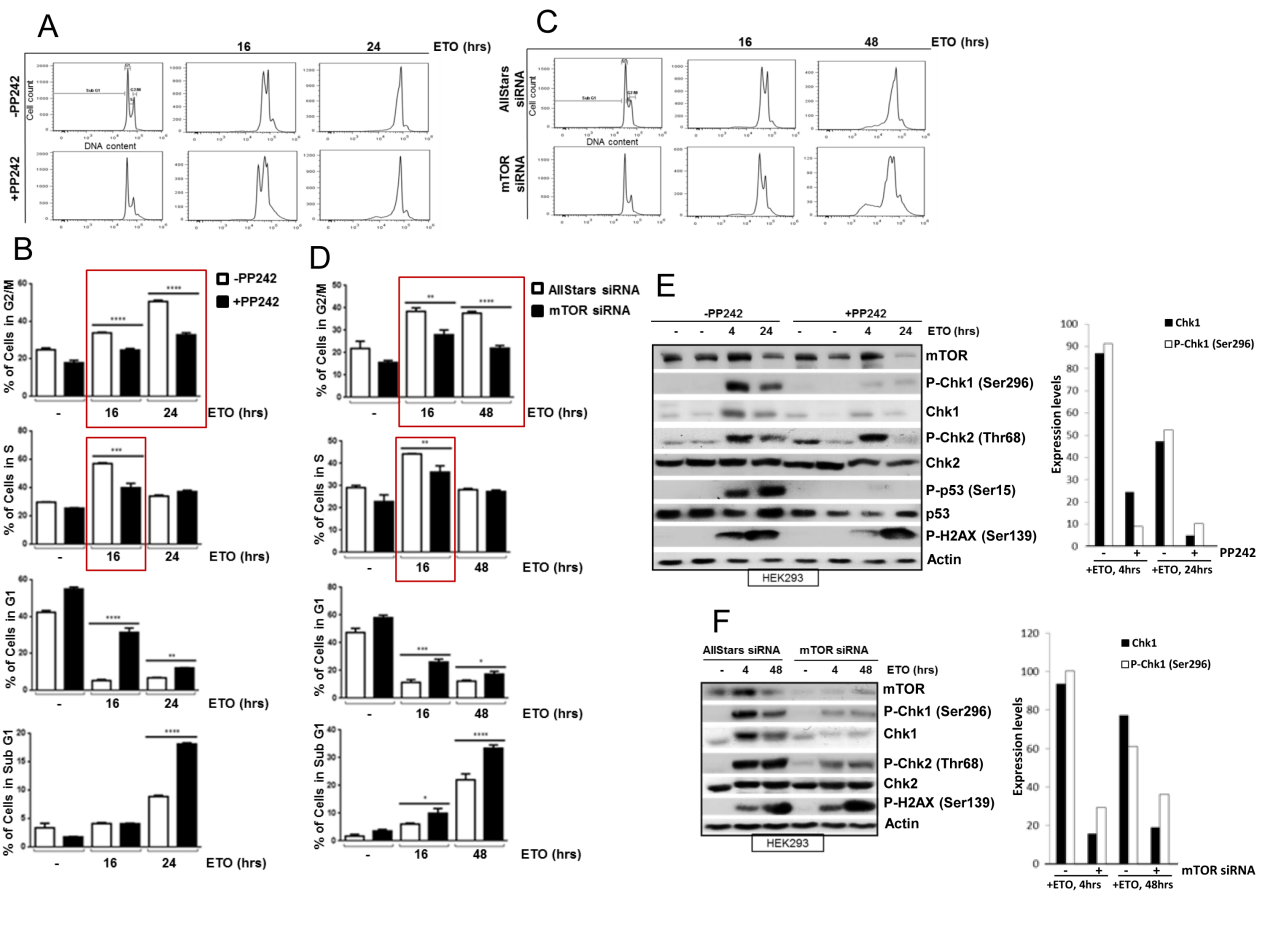
(A) mTOR is required for efficient DNA damage-induced S and G2/M cell cycle arrest. HEK293 cells were incubated in the absence or presence of 400 nM of PP242 for 1 hr before addition of 100 μM of etoposide for 16 and 24 hrs. Cells were fixed in ethanol and DNA was stained with propidium iodide and DNA content was assayed by flow cytometry (B) The percentage of cells in each cell cycle phase (G2/M, S, G1 and Sub G1) is shown. Bars represent mean ± SEM of three separate experiments. Statistical analysis was performed using two-way ANOVA with Bonferroni post-test. *P<0.05, **P<0.01, ***P<0.001, ****P<0.0001. (C) HEK293 cells were transiently transfected with AllStars control duplex or mTOR siRNA for 72 hrs. 100 μM of etoposide was added 16 and 48 hrs prior to the end of 72 hrs incubation period. Cells were fixed in ethanol and DNA was stained with propidium iodide and DNA content was determined by flow cytometry. (D) The percentage of cells in each cell cycle phase (G2/M, S, G1 and Sub G1) is shown. Bars represent mean ± SEM of three separate experiments. Statistical analysis was performed using two-way ANOVA with Bonferroni post-test. *P<0.05, **P<0.01, ***P<0.001, ****P<0.0001. (E) HEK293 cells were incubated in the absence or presence of 400 nM of PP242 for 1hr before addition of 100 μM of etoposide for 4 and 24 hrs. Whole-cell lysates were assayed by western blot for mTOR, Chk1 and phosphorylated Chk1 (Ser296), Chk2 and phosphorylated Chk2 (Thr68), p53 and phosphorylated p53 (Ser15), and phosphorylated histone H2AX (Ser139). Actin was used as a loading control. Chk1 protein and phosphorylated Chk1 (Ser296) expression levels were determined by densitometry and presented on a column graph. (F) HEK293 cells were transiently transfected with AllStars siRNA control duplexes or mTOR siRNA for 72 hrs. 100 μM of etoposide was added at 4 and 48 hrs prior to the end of 72 hrs incubation period. Whole-cell lysates were assayed by western blot for mTOR, Chk1 and phosphorylated Chk1 (Ser296), Chk2 and phosphorylated Chk2 (Thr68), and phosphorylated histone H2AX (Ser139). Actin was used as loading control. Chk1 protein and phosphorylated Chk1 (Ser296) expression levels were determined by densitometry and presented on a column graph.

The central role of the DNA damage response is to enhance cell survival. This is achieved by a coordinated response to DNA damage that delays cell cycle progression in order to maximize DNA repair. As mTOR is a key regulator of the cell cycle [27], we next assessed whether mTOR enhanced cell survival in response to DNA damage by promoting cell cycle arrest. DNA damage was induced in HEK293 cells with etoposide for 16 or 24 hrs in the absence or presence of an ATP competitive inhibitor of mTOR, PP242, which inhibits both mTORC1 and mTORC2 complexes [28], and the percentage of cells in different phases of the cell cycle was analysed by flow cytometry (Figure 3A and 3B). In the absence of PP242, efficient S and G2/M arrest was observed following etoposide treatment in HEK293 cells (Figure 3A and B). Importantly, a significant inhibition of cell cycle arrest was observed when mTOR activity was attenuated with PP242 (Figure 3A and B). Furthermore, siRNA-mediated downregulation of mTOR also led to a striking inhibition of both S and G2/M cell cycle arrest (Figure 3C and 3D). Taken together, these results show that mTOR is required for efficient DNA damage-induced S and G2/M cell cycle arrest.

### mTOR is required for DNA damage-induced Chk1 activation

**Figure 4:**
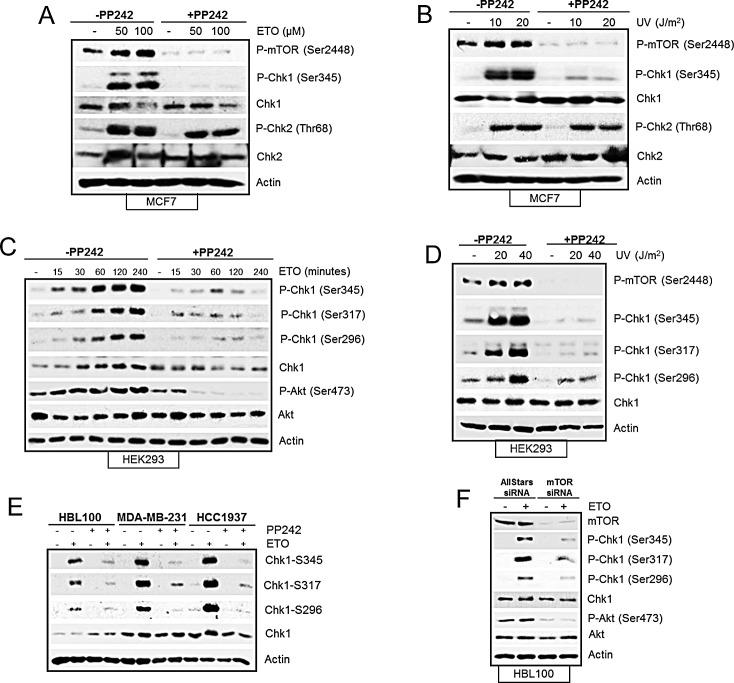
(A) Pharmacological inhibition of mTOR suppresses etoposide-induced Chk1 activation not Chk2. MCF7 cells were treated in the absence or presence of 400 nM PP242 for 1 hr before addition of 50 μM and 100 μM etoposide for 4 hrs. Whole-cell lysates were analyzed by western blot for phosphorylated mTOR (Ser2448), Chk1 (Ser345), and Chk2 (Thr68) and total protein levels of Chk1 and Chk2. Actin was used as a loading control. (B) Pharmacological inhibition of mTOR suppresses UV-induced Chk1 activation not Chk2. MCF7 cells were exposed to 10 and 20 joules of UV and left to recover in the presence of 400nM of PP242 for 4hrs. Whole-cell lysates were analyzed by western blot for phosphorylated mTOR (Ser2448), Chk1 and phosphorylated Chk1 (Ser345), Chk2 and phosphorylated Chk2 (Thr68). Actin was used as a loading control. (C) PP242 prevents etoposide-induced Chk1 phosphorylations and Chk1 protein level. HEK293 cells were incubated with 50 μM of etoposide in the absence and presence of 200 nM of PP242 for the time points indicated. Whole-cell lysates were assayed by western blot for Chk1 and phosphorylated Chk1 (Ser345, Ser296 and Ser317), Akt and phosphorylated Akt (Ser473). Actin was used as loading control. (D) PP242 prevents UV-induced Chk1 phosphorylations but not Chk1 protein level. HEK293 cells were exposed to 10 and 20 joules of UV and left to recover in the absence and presence of 400nM of PP242 for 2hrs. Whole-cell lysates were assayed by western blot for phosphorylated mTOR (Ser2448), Chk1 and phosphorylated Chk1 (Ser345, Ser296 and Ser317). Actin was used as loading control. (E) PP242 prevents etoposide-induced Chk1 phosphorylations in breast cancer cell lines. HBL100, MDA-MB-231 and HCC1937 cells were treated in the absence or presence of 400 nM PP242 for 1 hr before addition of 50 μM etoposide for 4 hrs. Whole-cell lysates were analysed by western blot for Chk1 and phosphorylated Chk1 (Ser345, Ser317 and Ser296). Actin was used as a loading control. (F) Ablation of mTOR with siRNA inhibits etoposide-induced Chk1 phosphorylations but not Chk1 protein in HBL100 cells. HBL100 cells were transiently transfected with AllStars control siRNA duplexes or siRNA to mTOR for a total of 72 hr. 50 μM of etoposide was added 4 hr before the end of the 72 hrs period. Whole-cell lysates were analysed by western blot for mTOR, Chk1 and phosphorylated Chk1 (Ser345, Ser317 and Ser296), Akt and phosphorylated Akt (Ser473). Actin was used as a loading control.

Key regulators of the DNA damage-induced cell cycle arrest include Chk1, Chk2 and p53 proteins [29]. We therefore assessed whether mTOR is required to activate these proteins by observing the status of the DNA damage-induced phosphorylation and their total protein levels. Western blot analysis revealed that etoposide-induced increase in phosphorylation and total protein level of Chk1, and p53 were reduced by pharmacological inhibition of mTOR kinase at 4 and 24 hrs. Although Chk2 phosphorylation was unaffected at 4hrs, it was reduced at 24 hrs (Figure 3E). To confirm that the effects we observed were specific to mTOR, we downregulated mTOR with siRNA and found that etoposide-induced phosphorylation of Chk1 and Chk2 were reduced as well as total Chk1 level (Figure 3F). Overall, DNA damage-induced phosphorylation of the histone protein, H2AX, a key indicator of the amount of damaged DNA, did not appear to be affected by mTOR inhibition (Figure 3E and F). In conclusion, these results show that mTOR is required for efficient DNA damage-induced cell cycle arrest, and this is possibly mediated by regulation of key cell cycle proteins Chk1, Chk2 and p53. mTOR-dependent induction of p53 after DNA damage has previously been reported [16, 18, 24, 30] and as HEK293 cells used here are known to have non-functional p53 [31], we further assessed mTOR-dependent regulation of Chk1 and Chk2 after DNA damage. We also extended our studies to include breast cancer cells as mTOR is emerging as an important target for breast cancer treatment. Pharmacological inhibition of mTOR with PP242 inhibited early etoposide- and UV-induced Chk1 phosphorylation in MCF7 cells, but not Chk2 phosphorylation (Figure 4A and 4B). In addition, siRNA-mediated downregulation of mTOR reduced total Chk1 level and phosphorylation, but not Chk2 phosphorylation, when a lower concentration of etoposide was used (Supplementary Figure 2). Therefore we decided to focus primarily on how mTOR is required for Chk1 regulation following early DNA damage.

It was evident from Figure 3 (E and F) that mTOR inhibition with PP242 or siRNA caused a reduction in total Chk1 protein and its phosphorylation, following etoposide-induced DNA damage in HEK293 cells. To dissect the mechanism of how mTOR regulates Chk1 we observed Chk1 under various conditions and in different cell lines. First we assessed the status of phosphorylation residues and total Chk1 level in HEK293 cells following etoposide-induced DNA damage from 15 minutes to 4hrs (Figure 4C). Following DNA damage, Chk1 is phosphorylated at Ser345 and Ser317 leading to autophosphorylation at Ser296 and Chk1 activation [32, 33]. As expected, etoposide-induced phosphorylation of these three residues of Chk1 from 15 minutes to 4 hrs (Figure 4C), all of which were inhibited in the presence of PP242 treatment (Figure 4C). In addition, as previously observed in HEK293 cells, PP242 also suppressed total Chk1 level following etoposide-induced DNA damage (Figure 4C). However, when ultraviolet (UV) irradiation was used as the DNA damaging agent, all three UV-induced Chk1 phosphorylations were reduced by PP242 in HEK293 cells without affecting total Chk1 protein level (Figure 4D). PP242 also prevented UV-induced Chk1 phosphorylation (Ser345) in MCF7 cells without affecting the total Chk1 level (Figure 4B). Furthermore, in other breast cancer cell lines (HBL100, MDA-MB-231 and HCC1937), PP242 (Figure 4E) and mTOR siRNA treatment in HBL100 cells (Figure 4F) inhibited all three Chk1 phosphorylations induced by etoposide. Interestingly, similar to HEK293 cells, mTOR inhibition caused a reduction in total Chk1 level following etoposide treatment in HCC1937 cells but not in HBL100 and MDA-MB-231 cell lines (Figure 4E and F). Collectively these results show that in all cell lines used in this study and by two different types of DNA damage induction, and two different types of mTOR inhibition, all three DNA damage-induced phosphoryations of Chk1 require mTOR activity. In addition, the total level of Chk1 also requires mTOR but in a cell-specific manner and depending on the type of DNA damage induction. Taken together these results demonstrate that mTOR is required for DNA damage induced Chk1 activity.

### mTOR regulates Chk1 production following etoposide-induced DNA damage

**Figure 5:**
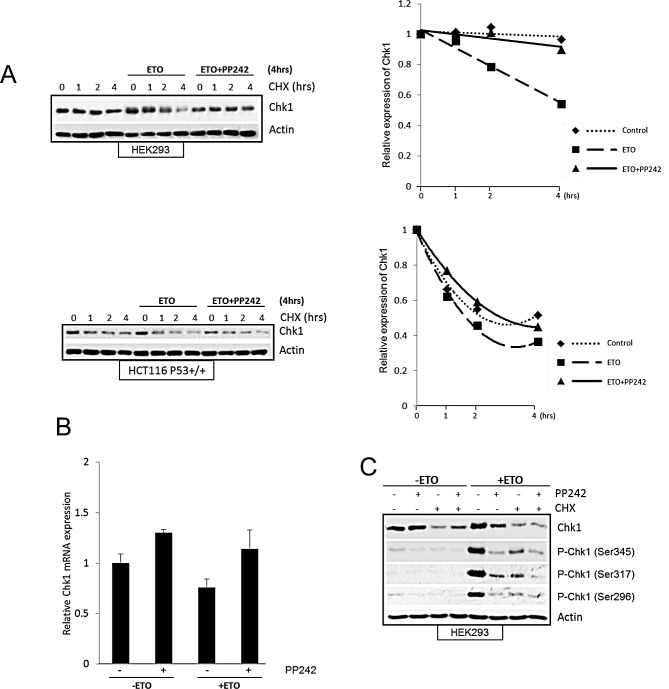
(A) mTOR inhibition does not decrease Chk1 half-life following DNA damage. HEK293 and HCC116 (p53^+/+^) cells were treated with 100μM etoposide or 400nM PP242+100μM etoposide for 4hrs, prior to this end 10 μM cycloheximide (CHX) was added for 1, 2 and 4hrs. As control cycloheximide alone was added for 1, 2 and 4hrs. Whole-cell lysates were analysed by western blot for Chk1. Actin was used as a loading control. Chk1 protein was determined by densitometry and normalised to 0 hr control, which is set as 1. (B) Pharmacological inhibition of mTOR does not affect Chk1 mRNA level after DNA damage. HEK293 cells were treated in the absence or presence of 400 nM PP242 for 1 hr before addition of 100 μM etoposide for 4 hrs. mRNA expression of Chk1 was assessed by real-time PCR relative to GAPDH. Mean±S.E. of duplicate values of one representive experiment shown. (C) mTOR inhibition does not cause further decrease in Chk1 protein in the presence of translation inhibitor after DNA damage. HEK293 cells were pre-treated with 10μM of cycloheximide, or 400nM PP242, or together for 1hr followed by 100μM etoposide for further 4 hrs. As controls cells were treated with 100μM etoposide for 4hrs, or 10μM cycloheximide, 400nM PP242 or together for 5 hrs. Whole-cell lysates were assayed by western blot for Chk1 and phosphorylated Chk1 (Ser345, Ser317 and Ser296). Actin was used as loading control.

Since mTOR inhibition in HEK293 cells significantly reduced the total Chk1 level following etoposide treatment (Figure 3), we explored how mTOR regulates Chk1 protein in these cells. The reduction in Chk1 level caused by mTOR inhibition could be due to faster degradation of Chk1 or inhibition of its production at transcriptional or translational level. Therefore, we first observed the half-life of Chk1 using cycloheximide. In agreement with past reports [34-36] the turnover of Chk1 protein was significantly increased by etoposide-induced DNA damage in both HEK293 and HCT116 cells (Figure 5A). mTOR inhibition with PP242 following DNA damage did not further increase Chk1 turnover, therefore it is unlikely that the decrease in Chk1 caused by mTOR inhibition is due to an increase in Chk1 degradation. Unexpectedly, PP242 in fact reduced Chk1 turnover following DNA damage. Zhang [34] demonstrated that DNA damage induced phosphorylation of Chk1 at Ser345 targets it for ubiquitin-mediated proteasomal degradation. Since we observed that PP242 inhibited Chk1 phosphorylation at Ser345, this could account for why Chk1 degradation is prevented. Nevertheless, total Chk1 is still reduced by mTOR inhibition following etoposide-induced DNA damage. Therefore, these results indicate that mTOR inhibition causes Chk1 reduction by inhibiting its production. Next we measured Chk1 mRNA levels using RT-PCR and found that they were not changed by etoposide-induced DNA damage, nor by mTOR inhibition with PP242 (Figure 5B). Thereby showing that mTOR regulation of Chk1 protein production is not mediated through transcription. However, in the presence of cycloheximide Chk1 level is efficiently suppressed before and after DNA damage, more importantly PP242 did not cause a further reduction in Chk1 (Figure 5C) implying that Chk1 reduction caused by mTOR inhibition is mediated by preventing its synthesis at translation level. These results collectively suggest that following etoposide-induced DNA damage mTOR regulates Chk1 production through protein synthesis. Figure 5C further supports our concept that mTOR is required for Chk1 phosphorylation and activation independently from its regulation of total Chk1 protein. In the presence of cycloheximide, total Chk1 is suppressed but not further reduced by PP242. In the presence of cycloheximide, all three etoposide-induced Chk1 phosphorylations are reduced but PP242 causes additional reduction.

### mTORC2 complex is required for etoposide-induced activation of Chk1

**Figure 6:**
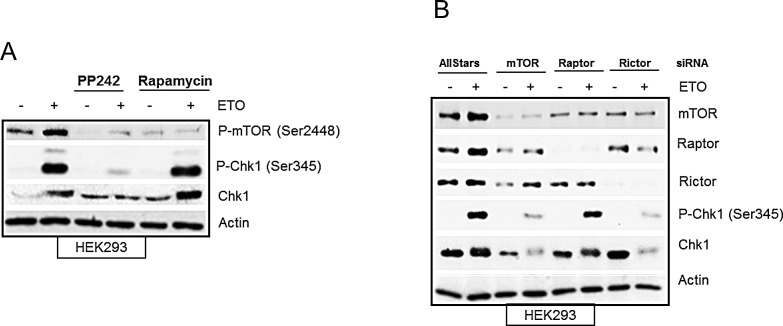
(A) Etoposide-induced Chk1 activation is independent of mTORC1. HEK293 cells were treated in the absence or presence of 400 nM of PP242 or 100 nM of rapamycin for 1 hr before addition of 100 μM of etoposide for 4 hrs. Whole-cell lysates were assayed by western blot for phosphorylated mTOR (Ser2448), Chk1 and phosphorylated Chk1 (Ser345). Actin was used as a loading control. (B) Etoposide-induced Chk1 activation is dependent on mTORC2. HEK293 cells were transiently transfected with AllStars control duplexes or siRNA against mTOR, Raptor or Rictor for 72 hrs. 50 μM of etoposide was added 4 hrs prior to the end of 72 hrs incubation period. Whole-cell lysates were assayed by western blot for protein levels of mTOR, Raptor, Rictor, Chk1 and phosphorylated Chk1 (Ser345). Actin was used as loading control.

In mammalian cells, mTOR forms two functionally distinct complexes, mTORC1 and mTORC2, which contain shared and distinct partners. While mTORC1 exclusively contains a scaffolding protein, Raptor, required for its function [37] mTORC2 complex contains Rictor, needed for its assembly [10]. PP242 inhibits both mTORC1 and mTORC2 complexes, therefore in order to dissect out the contribution of mTORC1 and/or mTORC2 to DNA damage mediated Chk1 regulation we used rapamycin, which predominantly inhibits mTORC1, as well as specific downregulation of Raptor and Rictor with siRNA. Rapamycin had no effect on early etoposide-induced Chk1 phosphorylation and protein level as compared with PP242 (Figure 6A), suggesting that mTORC1 activity was dispensable for DNA damage mediated regulation of Chk1. Instead these data suggested a requirement of mTORC2 for etoposide-induced Chk1 activation as siRNA against Raptor (selective downregulation of mTORC1) did not affect Chk1, whereas siRNA against Rictor (selective downregulation of mTORC2) did prevent etoposide-induced Chk1 phosphorylation and total Chk1 protein level (Figure 6B). These results are in line with recent work suggesting an increasing role of mTORC2 in cell cycle progression [38]. Taken together, these results show that early etoposide-induced increase in Chk1 phosphorylation and total Chk1 protein was dependent on mTORC2.

### mTORC1/2 inhibition sensitizes breast cancer cells to chemotherapy

**Figure 7:**
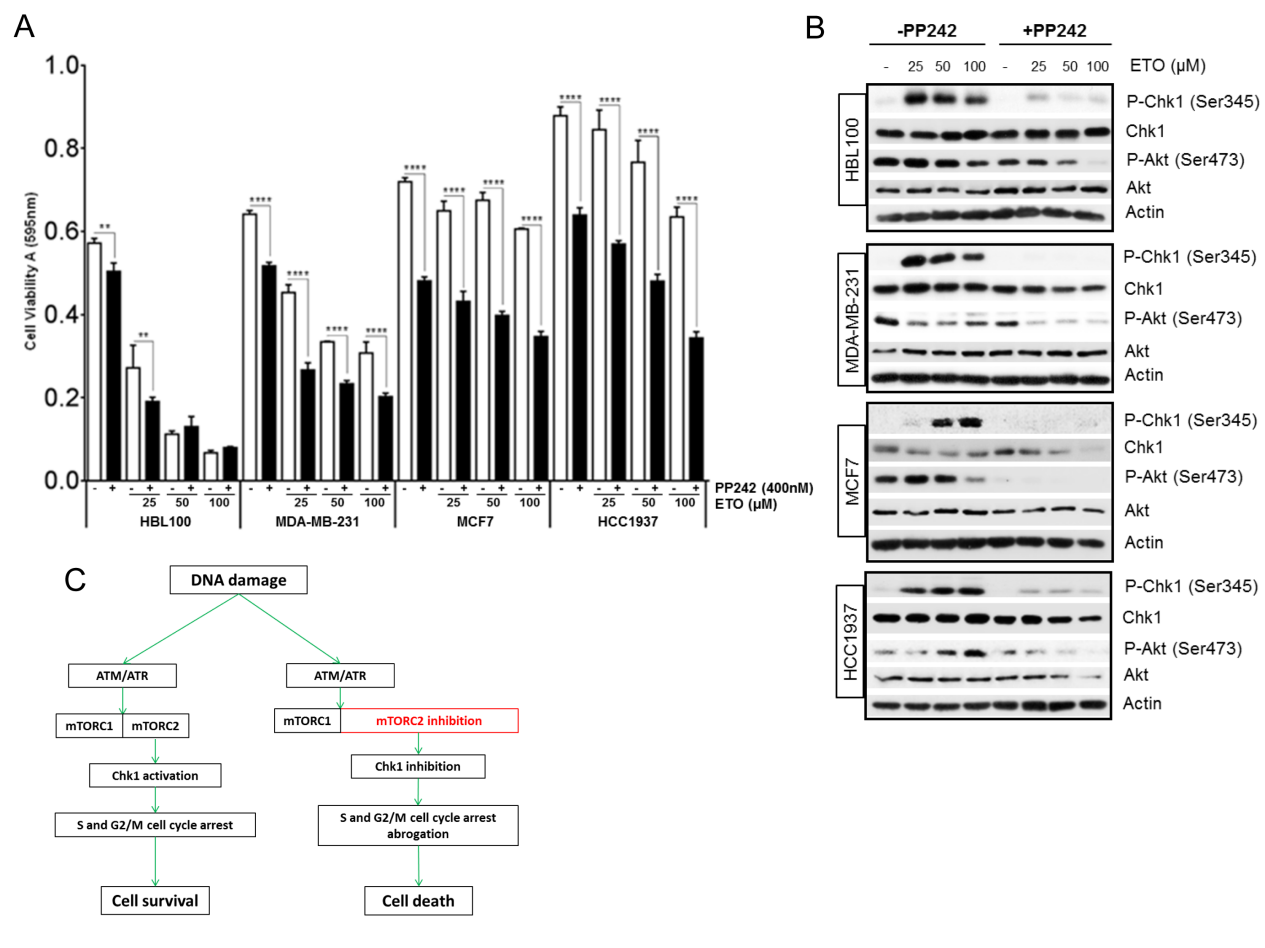
(A) Pharmacological inhibition of mTORC1/2 sensitizes breast cancer cells to chemotherapy-mediated cell death. HBL100, MDA-MB-231, MCF7 and HCC1937 breast cells were seeded at 1.5×10^4^ cells/cm^2^ in 96-well plates and incubated in the absence or presence of 400 nM of PP242 for 1 hr, before addition of etoposide at the concentrations indicated for 24 hrs. Cell viability was assessed by MTT assay. Bars represent the mean ± SEM of three separate experiments. Statistical analysis was performed using two-way ANOVA with Bonferroni post-test. *P<0.05, **P<0.01, ***P<0.001, ****P<0.0001. (B) Pharmacological inhibition of mTOR suppresses etoposide-induced Chk1 activation in breast cancer cells. HBL100, MDA-MB-231, MCF7 and HCC1937 breast cells were incubated in the absence or presence of 400 nM of PP242 for 1 hr, before addition of etoposide at the concentrations indicated for 24 hrs. Whole-cell lysates were assayed by western blot for Chk1 and phosphorylated Chk1 (Ser345), Akt and phosphorylated Akt (Ser473). Actin was used as loading control. (C) Proposed model for mTORC2 regulation of the DNA damage response. A transient increase in mTORC2 activity after DNA damage by ATM/ATR contributes to the activation of Chk1 and efficient S and G2M cell cycle arrest which allows more time for DNA repair and cell survival. Consequently, when mTORC2 is inhibited Chk1 activation and cell cycle arrest is prevented and the time for repair is removed, which allows DNA damage to induce cell death more effectively.

mTOR inhibitors can either sensitize cells to chemotherapy or attenuate the ability of chemotherapeutics to induce apoptosis through multiple mechanisms which are not yet fully elucidated but seem to depend, at least in part, on the genetic context of cells. For example, the rapalog everolimus, sensitized lung carcinoma cells to cisplatin treatment [15], whereas in colon cancer and renal carcinoma cell lines, pharmacological inhibition of mTOR kinase prevented chemotherapy-induced cell death [24, 39]. In HEK293 cells, the inhibition of mTOR activity using both PP242 and siRNA led to an increase in etoposide-induced cell death, as evidenced from the increase in the sub G1 population (Figure 3B and 3D). In breast cancer, the mTOR signalling pathway is commonly dysregulated and is implicated in resistance to current treatment [40, 41]. We analysed a panel of breast cancer cell lines to assess cell viability following etoposide-induced DNA damage (Figure 7A). One cell line, HBL100, an immortalized epithelial cell line, displayed high sensitivity to etoposide as compared with three other breast cancer cell lines, MDA-MB-231, MCF7 and HCC1937, which demonstrated varying degrees of resistance to etoposide (Figure 7A). Importantly, this resistance was overcome by the inhibition of mTOR activity with PP242, which significantly decreased breast cancer cell viability following DNA damage (Figure 7A). Consistent with our previous results, western blot analysis revealed that etoposide-induced Chk1 phosphorylation was strikingly inhibited by PP242 in all breast cell lines tested (Figure 7B). Interestingly the total Chk1 protein level was also reduced by PP242 following DNA damage in these cells with the exception of HBL100 (Figure 7B). The mTORC2-specific phosphorylation of Akt at Ser473 was also monitored by western blot to confirm that mTORC2 activity was sufficiently inhibited by PP242 in these cell lines. Collectively, these results demonstrate that inhibition of mTOR activity significantly potentiates etoposide-mediated cell death in breast cancer, suggesting that breast cancer cells may rely on the mTORC2-Chk1 pathway for survival. In line with this, recent work has demonstrated that cisplatin-induced apoptosis was significantly increased by loss of Rictor but not Raptor in breast and ovarian cancer cells [40, 42].

## DISCUSSION

Since its discovery as the target of rapamycin, mTOR has been identified as a crucial mediator of protein synthesis, cell growth, and metabolism. mTORC1 is also important for relaying signals to the cell machinery in response to DNA damage. A number of studies have demonstrated that mTORC1 is downregulated in response to DNA damage in a p53 dependent manner [13, 14]. However, others have reported an increase in mTOR kinase activity in response to DNA damage [16, 19-21]. The mechanism by which mTOR promotes cell survival under conditions of DNA damage is poorly understood. Here, we found an increase in both mTORC1 and mTORC2 activity induced by early DNA damage. In addition, mTOR protein levels were transiently increased by etoposide treatment in several cell lines. This observed increase was not due to increased protein synthesis, but likely due to a transient stabilization of mTOR that was dependent on ATM and ATR and independent of p53. As mTOR is a key regulator of cell cycle progression from G1 to S phase [27], we investigated whether mTOR promotes cell survival following DNA damage by regulating cell cycle arrest, which would consequently allow more time for DNA repair. Indeed, our results show that inhibition of mTOR by both siRNA and a specific inhibitor caused a striking inhibition of etoposide-induced S and G2/M cell cycle arrest. Further analysis revealed that the activation of a key cell cycle arrest regulator Chk1 was dependent on mTOR, as mTOR inhibition prevented etoposide-induced increase of Chk1 phosphorylation and total protein level. mTOR inhibition with siRNA, against mTOR or Rictor, or with PP242 consistently caused a reduction in Chk1 phosphorylation in all cell lines used in this study and by two different types of DNA damage, thereby demonstrating a requirement for mTOR in Chk1 activation following DNA damage. In addition, mTOR inhibition caused a reduction in Chk1 protein level under certain conditions, particularly in HEK293 cells after etoposide-induced DNA damage. Since Chk1 is degraded faster after DNA damage and we found no change in mRNA expression, mTOR most likely regulates Chk1 at translational level. The mechanism of how mTOR regulates Chk1 phosphorylation remains to be investigated. ATR is known to directly phosphorylate and activate Chk1, however we found ATR to be upstream of mTOR. It will be interesting in future studies to identify if mTOR or one of its downstream targets are responsible for Chk1 phosphorylation, or whether mTOR is involved in the regulation of Chk1 phosphatases [43].

In this study we show that mTORC2 and not mTORC1 regulates Chk1 following DNA damage. A previous report has demonstrated that mTORC2 regulates cell survival following DNA damage as glioblastoma cells with elevated mTORC2 activity were resistant to chemotherapy which was overcome by mTORC2 inhibition [44]. In addition, TORC2 mediated actin filament regulation to promote cell survival following low DNA damage in yeast [45]. Our work is the first to link mTORC2 to Chk1 regulation in DNA damage response signalling to promote cell survival.

We also observed an effect of mTOR inhibition on the DNA damage-dependent phosphorylation of Chk2 (Figure 3E and 3F). However, this effect was only observed following late DNA damage (24 h after etoposide treatment) which suggests that mTOR-dependent regulation of the S and G2/M cell cycle arrest is mainly dependent on Chk1 and not Chk2. However, this does not exclude a role for mTOR-dependent regulation of Chk2 in the DNA damage response as a recent report shows that in response to DNA damage, mTORC1 through FANCD2 is required for ATM-Chk2 activation in rhabdomyosarcoma cells [22].

mTOR signalling is commonly activated in cancer which has led to the generation of a number of mTORC1 inhibitors (rapalogs) that have demonstrated clinical efficacy in a subset of cancers including relapsed renal cell carcinoma [46] and postmenopausal hormone-receptor-positive breast cancer [47]. It has become increasingly clear that mTORC1 and mTORC2 exert distinct cellular functions, and that combined inhibition of both complexes may fully exploit the anti-cancer potential of targeting mTOR. Indeed, in a panel of breast cancer cell lines, cell survival was significantly decreased when etoposide was combined with pharmacological inhibition of mTORC1/2, demonstrating that mTORC1/2 inhibitors are able to sensitize breast cancer cells to chemotherapy, consistent with a previous study [40]. An important question for the clinical development of mTOR inhibitors is why ablation of mTOR kinase sensitizes some cancer cells to DNA damage-induced cell death, but has the opposite effect in other cell types. For example, we and others have shown that mTOR inhibition attenuates chemotherapy-mediated cell death in colon and renal cell carcinoma cell lines [24, 39], and in certain genetic contexts, such as loss of TSC1/2 [18] or REDD1 [17]. The molecular mechanisms underlying these differential effects of mTOR inhibition in different cellular contexts is poorly understood, but is likely to depend on multiple pathways. One possibility is that the p53 status of cells is crucial, since loss of TSC1/2 or REDD1 leads to hyperactive mTOR and increased p53 translation [17, 18]. Consequently, in cells that undergo DNA damage-induced p53-dependent cell death, mTOR ablation could prevent p53-mediated cell death. However, in cells that depend on alternative apoptotic pathways and/or rely on mTORC2-Chk1 for cell cycle arrest, then by preventing appropriate cell cycle checkpoints, mTOR inhibition can augment cell death. While further studies are required to delineate the underlying mechanisms, collectively, these data highlight the need for careful evaluation of the genetic context of cells in order to fully exploit the use of targeted mTOR therapeutics.

We could consistently show that DNA damage-induced Chk1 activation was dependent on mTOR in all cell lines studied, suggesting that cells may rely on mTOR-Chk1 signalling for survival. Numerous studies have demonstrated that Chk1 inhibition following DNA damage potentiates DNA damage-induced cell death via multiple mechanisms [48-53]. Importantly, this study has revealed an unexpected benefit of mTORC1/2 inhibitors in their ability to inhibit Chk1 activity and cell cycle arrest. We show reduced cell survival when mTORC1/2 is inhibited in the presence of genotoxic stress and report that mTORC2 is essential for Chk1 activation. Our data provides new mechanistic insight into the role of mTOR in the DNA damage response and support the clinical development of mTORC1/2 inhibitors in combination with DNA damage-based therapies for breast cancer.

## MATERIALS AND METHODS

### Reagents

Etoposide, PP242 and rapamycin were purchased from Sigma Aldrich, Gillingham, UK. ATM specific inhibitor (# 118500), cycloheximide and MG-132 were all purchased from Merck Millipore, Watford, UK.

### Cell culture

All cell lines were grown at 37°C and 5% CO_2_ and maintained in Dulbecco's modified Eagle medium (PAA Laboratories, Yeovil, UK) supplemented with 10% fetal bovine serum (Sigma-Aldrich), 100 IU/mL penicillin, 100 μg/mL streptomycin and 2 mM glutamine and 1% Fungizone amphotericin B (all purchased from Life Technologies, Paisley, UK). Matched human colorectal carcinoma cells (HCT116 p53^+/+^ and p53^−/−^) were kindly provided by Professor Galina Selivanova (Karolinska Institute, Stockholm, Sweden). HBL100 and MDA-MB-231 cell lines were a gift from Dr Kay Colston (St George's, University of London, UK).

HEK293, MCF7 and HCC1937 cells were obtained from American Type Culture Collection (Manassas, VA, USA).

### UV-irradiation

Cells were seeded in 6 cm dishes and grown to 50-70% confluence. Medium was removed, replaced with PBS and cells were exposed to ultraviolet radiation (UV) for various time points. PBS was aspirated and replaced with DMEM containing 10% FBS and left to recover for various times. Thereafter, whole-cell lysates were assayed by western blot as previously described [54].

### Gene silencing with siRNAs

1.5×10^4^/cm^2^ HEK293 cells were seeded for 2-4 hrs until attached. Cells were transiently transfected with 25 nM of siRNA duplexes using HiPerfect reagent (QIAGEN, Crawley, UK,) according to the manufacturer's instructions and as previously described [55]. mTOR siRNA (#6381) was purchased from Cell Signaling (New England Biolabs, Hitchin, UK), Raptor (sc-44069) and Rictor (sc-61478) siRNA duplexes were purchased from Santa Cruz (CA, USA). AllStars siRNA duplex (QIAGEN) was used as negative control. Following addition of siRNA duplexes, etoposide was added at 4, 16 or 48 hrs prior to the end of a total 72 hrs in siRNA and cells were harvested on ice for flow cytometry and western blot analysis.

### Real-time quantitative PCR

Total RNA was extracted from cells using the RNeasy Mini Kit (QIAGEN) and 50 ng total RNA was used for first-strand cDNA synthesis using Sensiscript (QIAGEN) according to the manufacturer's instructions. Real-time qPCR was performed with GoTaq qPCR master mix (Promega, Southampton, UK) in a Stratagene Mx3000 real time cycler (Agilent Technologies, Stockport, UK). Primers for Chk1 were purchased from Sigma-Aldrich (forward 5′-GGTGCCTATGGAGAAGTTCAA; reverse 5′-TCTACGGCACGCTTCATATC) [56]. Primers for GAPDH have been described previously [55].

### Western blotting

Experiments with cell lines were carried out as indicated in the figure legends and cells were lysed in 2×Laemmli buffer and protein concentration was determined using the Lowry assay, as previously described [54]. Proteins were separated by SDS-PAGE and transferred on to PVDF to be probed with specific antibodies. Actin was purchased from Merck Millipore; all other antibodies were from Cell Signaling, NEB.

### Flow cytometry

Flow cytometry was performed as previously described [24]. Following treatment, cells were harvested on ice and fixed in 70% ethanol for at least 1 hr at −20°C. Thereafter, cells were washed in PBS and resuspended in 50 μg/mL propidium iodide/RNAse A solution (0.5 mL per 10^6^ cells) (Sigma-Aldrich) for 30 min in the dark at room temperature. The percentage of cells in different phases of cell cycle was analysed with a Beckman Coulter Cytomics FC500 flow cytometer.

### MTT cell viability assay

Cells were seeded in 96-well plates and treated as indicated in the figure legends. Thereafter, 25 μl of MTT (3-[4,5-dimethylthiazol-2-yl]-2,5 diphenyl tetrazolium bromide) (Sigma-Aldrich) reagent was added to each well and incubated at 37°C for 2 hrs. Cells were lysed by addition of 100 μl of 10% SDS per well, and incubated at 37°C for 16 hrs before measurement of absorbance in a spectrophotometer (SpectraMax340PC384, Molecular Devices) at 595 nm.

### Statistical analysis

Two-way ANOVA was performed with Bonferroni multiple comparisons post-test that allowed determination of statistical significance between multiple samples simultaneously.

## SUPPLEMENTARY MATERIAL AND FIGURES


