# Supplementary material for: DNA damage-induced S and G2/M cell cycle arrest requires mTORC2-dependent regulation of Chk1
Source: Oncotarget. 2014 Nov 15;6(1):427–40. doi: 10.18632/oncotarget.2813 (PMC4381605; doi:10.18632/oncotarget.2813)
Supplement: Supplementary file 1 [file oncotarget-06-427-s001.pdf]

DNA damage-induced S and G2/M cell cycle arrest requires mTORC2-dependent regulation of Chk1

Supplementary Material

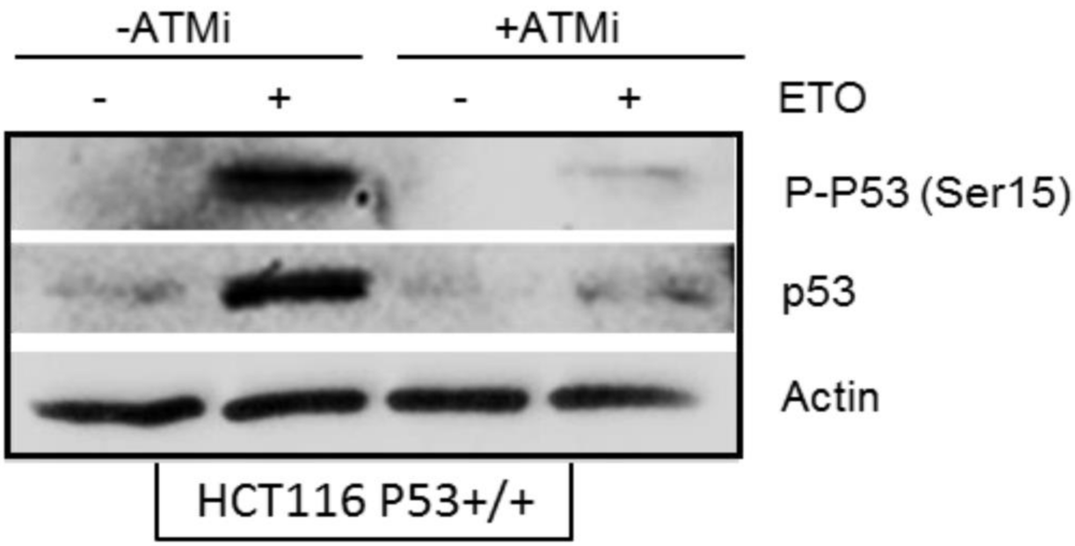

Supplementary Figure 1.
Pharmacological inhibition of ATM activity with ATMi prevents etoposide-induced accumulation and phosphorylation of p53, a well-known substrate of ATM. HCT116 $p53^{+/+}$ cells were pre-treated in the absence or presence of 10 μM ATM inhibitor (ATMi) for 1 hr before incubation with 100 μM etoposide for 4 hrs. Whole-cell lysates were assayed by western blot for p53 and phosphorylated p53 (Ser15). Actin was used as a loading control.

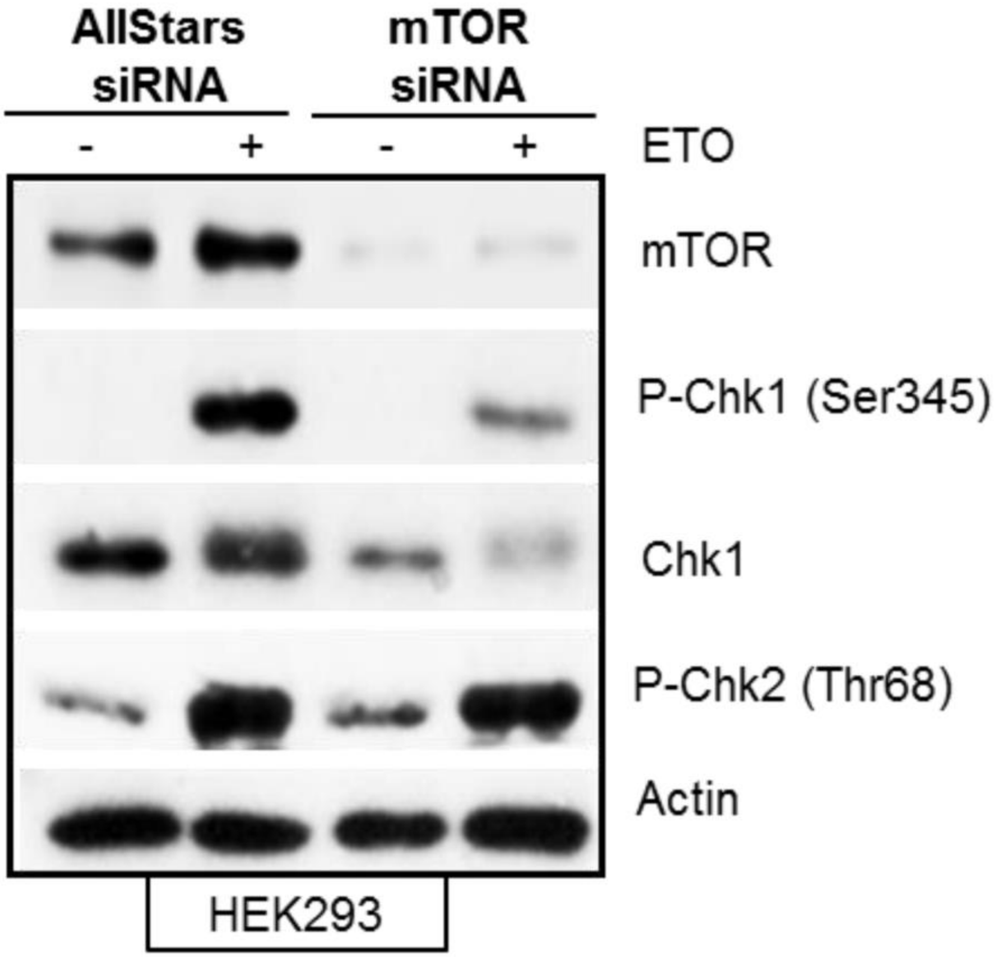

Supplementary Figure 2.
mTOR dowregulation with siRNA causes inhibition of Chk1 activation by lower amount of etoposide but not Chk2 activation. HEK293 cells were transiently transfected with AllStars siRNA control duplexes or mTOR siRNA for 72 hrs. 50 µM of etoposide was added at 4 hrs prior to the end of 72 hrs incubation period. Whole-cell lysates were assayed by western blot for mTOR, Chk1 and phosphorylated Chk1 (Ser296), and phosphorylated Chk2 (Thr68). Actin was used as loading control.
